# Antitumor activity of celastrol by inhibition of proliferation, invasion, and migration in cholangiocarcinoma via PTEN/PI3K/Akt pathway

**DOI:** 10.1002/cam4.2719

**Published:** 2019-11-26

**Authors:** Biqiang Zhu, Yunwei Wei

**Affiliations:** ^1^ Department of Oncology and Laparoscopy Surgery The First Affiliated Hospital of Harbin Medical University Harbin Heilongjiang China; ^2^ Translational Medicine Research and Cooperation Center of Northern China Harbin Heilongjiang China

**Keywords:** Akt, apoptosis, celastrol, epithelial‐to‐mesenchymal transition, phosphatase and tensin homolog

## Abstract

**Aim:**

Cholangiocarcinoma is a malignant tumor originating from bile duct epithelium. Currently, the treatment strategy is very limited and the prognosis is poor. Recent studies reported celastrol exhibits antigrowth and antimetastasis properties in many tumors. Our study aimed to assess the anti‐CCA effects of cholangiocarcinoma (CCA) and the mechanisms involved in it.

**Methods:**

In this study, the long‐term and short‐term antiproliferation effects was determined using colony formation and Cell Counting Kit‐8 (CCK‐8) assays, respectively. Flow cytometry was performed to quantify apoptosis. Furthermore, wound healing and transwell assays were performed to determine the cell migration and invasion capabilities, respectively. To further find the mechanism involved in the celastrol‐induced biological functions, LY204002, a PI3K/Akt signaling inhibitor, and an Akt‐1 overexpression plasmid were employed to find whether PI3K/Akt pathway was involved in the celastrol‐induced CCA cell inhibition. Additionally, short interfering RNA (siRNA) was also used to investigate the mechanism involved in the celastrol‐induced PI3K/Akt signaling inhibition. Western blotting and immunofluorescence assays were also performed to detect the degree of relative proteins. Moreover, we validated the antiproliferation and antimetastasis effects of celastrol in vivo by constructing subcutaneous and lung metastasis nude mice models.

**Results:**

We discovered that celastrol effectively induced apoptotic cell death and inhibited the capacity of migration and invasion in CCA cells. Further mechanistic study identified that celastrol regulated the PI3K/Akt signaling pathway, and the antitumor efficacy was likely due to the upregulation of PTEN, a negative regulator of PI3K/Akt. Blockage of PTEN abolished the celastrol‐induced PI3K/Akt signaling inhibition. Additionally, in vivo experiments conformed celastrol inhibited the tumor growth and lung metastasis with no serious side effects.

**Conclusions:**

Overall, our study elucidated a mechanistic framework for the anti‐CCA effects of celastrol via PTEN/PI3K/Akt pathway.

## Graphical abstract

Our research provided mechanistic insight into the anti‐CCA effect of celastrol by the regulation of the PTEN expression, and provide a novel therapeutic strategy for CCA.

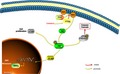

## 1 INTRODUCTION

Cholangiocarcinoma (CCA) is a rare primary hepatobiliary malignancy with a dismal prognosis arising from the epithelial cells of extrahepatic or/and intrahepatic biliary tree.^1^ An increasing number of researches demonstrated that the overall morbidity and mortality are increasing in recent years.^2^ Therapeutic options are limited because of its aggressive nature and often diagnosed at advanced stages, for which curative surgery is not a viable option.^3^ Curative surgical treatment is the only viable option that can provide the possibility of cure for locally restricted disease and prolonged survival. It is worth noting that radical resection may be associated with severe postoperative complications such as hepatorenal syndrome and progressive liver failure.^4^ In recent years, other nonsurgical treatment, such as molecular target therapy, adjuvant radiation therapy, systemic chemotherapy, and combination treatment have been improved for CCA therapy.^5^, ^6^, ^7^ Unfortunately, the overall treatment outcome of CCA is still limited. Thus, we urgently need new therapeutic strategies to provide optimism for CCA patients.

Celastrol, derived from the Chinese herbal medicine Trypterygium,^8^ exhibits extensive biological and pharmacological activities. Zhang J claimed that celastrol ameliortes inflammation on Human Retinal Pigment Epithelial Cells.^9^ An SY demonstrated that celastrol can significantly suppress microbial processes.^10^ According to the recent discovery, celastrol exhibits an antitumor effect on several cancer cells through inhibiting cell survival, invasion/migration, and angiogenesis or promoting cell apoptosis in a wide variety of tumor models, including osteosarcoma, colorectal cancer, liver cancer, lung cancer, and breast cancer.^11^, ^12^, ^13^ Mechanically, celastrol meditates tumor‐related proinflammatory cytokines, adhesion molecules, TGF‐activated kinase 1 (TAK1), NF‐kB, CXCR4, VEGF receptor (VEGFR), proteasome, and STAT3.^14^, ^15^, ^16^, ^17^, ^18^, ^19^, ^20^ Considering CCA is also an epithelial originated cancer like liver cancer or lung cancer, we hypothesized that celastrol may be an appropriate candidate for CCA treatment.

Although the exact mechanism leading to CCA has not been fully demonstrated, cumulative studies have shown that the phosphoinositide 3‐kinase (PI3K)/Akt plays an important role in the response of cells stimulated by external signals, and the abnormal activation of the PI3K/Akt pathway is closely related to the occurrence and development of malignant tumors, including CCA.^21^ Blocking multiple signaling pathways is the theoretical basis of many anticancer drugs. Surprisingly, celastrol has shown the potential to block PI3K/Akt signaling pathways in many anticancer studies. Pang X et al demonstrated that celastrol targeted Akt/mTOR/P70S6K signaling pathway in endothelial cells.^18^ Kannaiyan et al found that celastrol induced inhibition of proliferation vis suppression of PI3K/Akt signaling pathways in RPMI‐8226 cells.^22^ Considering PI3K/Akt signaling pathways act as an essential role in CCA development, we put forward the hypothesis that celastrol have the potential to inhibit CCA cell growth and metastasis.

In our study, the effects in CCA cells with regard to morphology, proliferation, migration, and invasion properties following celastrol treatment were examined. Mechanically, the blockage of PI3K/Akt ‐mediated signaling by upregulating PTEN is responsible for the changes. Our discoveries provided novel insights into celastrol as a promising anti‐CCA agent.

## 2 MATERIALS AND METHODS

### 2.1 Cells and culture

Human cholangiocarcinoma TFK‐1 and HuCCT‐1 cell lines were maintained in RPMI‐1640 medium containing 10% FBS and 1% penicillin‐streptomycin solution (all from Gibco) in an incubator with 5% carbon dioxide at 37°C.

### 2.2 Materials

Celastrol was purchased from Sigma, which was dissolved in dimethyl sulfoxide (DMSO) at the concentration of 100 mmol/L before use. LY294002 was purchased from Abcam Corporation (Cambridge, Cambridge shire, UK). The cytotoxic activity of celastrol on CCA cells was analyzed using CCK‐8 (Dojindo Molecular Technologies, Kimamoto). Primary antibodies were as followed (1:2000, CST): cleaved caspase‐3, 9, Bax, Bcl‐2, MMP‐2, 9, vimentin, E‐cadherin, PTEN, PI3K, Akt, p‐Akt, mTOR, p‐mTOR, Ki‐67, NF‐κB. In addition, β‐actin (1:1000, proteintech) was used as an internal control.

### 2.3 Cell viability

CCK‐8 assay was used to determine the cell viability according to the manufacturer's instructions. Briefly, after cells were seeded in a 96‐well plate and incubated with celastrol for indicated hours. The optical density (OD) values were used as the index of cell viability at the absorbance of 450 nm. Five replicate wells were set up in each group.

### 2.4 Colony formation assay

A total of 700/well cells were firstly seeded in 35‐mm dishes until most of them were attached to the bottom. Then, different concentrations of celastrol was used for each dish. After two weeks, the cells were fixed and stained. Colonies containing ≥ 20 cells were counted.

### 2.5 Apoptosis assay

Apoptotic degree of cells was measured by Annexin V‐FITC kit (BD Pharmingen). Cells in each group were firstly digested by trypsin without EDTA. Subsequently, the collected cells were resuspended with binding buffer (500 µL) and annexin V–FITC (5 µL) for 30 minutes. After that, PI (10 µL) was added in dark for 10 minutes at room temperature. Finally, the percentage of apoptotic cells were determined using BD Accuri^TM^ C6 flow cytometer (Becton‐Dickinson). Each experiment was conducted for three times.

### 2.6 Wound healing assay

A total of 5 × 10^4^ cells was initially seeded in each well of a 6‐well plate for 24 hours. Cell layer was scratched by a pipette tip (200 µL). Subsequently, cells were then continued cultured with indicated concentrations of celastrol. Representative wound was imaged by an optical microscope. Each experiment was conducted for three times.

### 2.7 Transwell assay

The experiment was conducted using 24‐well transwell chambers with 50 µL of Matrigel (2.0 mg/mL, BD Biosciences), and the cell invasive ability is assessed by the number of cells passing through the chambers. Cells (2.5 × 10^4^) were initially suspended with or without celastrol into the upper chamber, and the rest of the steps are the same as our previous reports.^23^

### 2.8 Immunofluorescence assay

Cells were initially fixed with paraformaldehyde (4%) for 20 minutes at room temperature. To increase cell membrane permeability, cells were also incubated with goat serum (10%) and Triton (1%). Subsequently, cells were incubated with antibodies for p‐Akt (Thr308) (1:200), p‐Akt (Ser437) (1:200) or PTEN (1:200) antibodies overnight at 4°C. After that, cells were incubated with an Alexa Fluor‐conjugated IgG secondary antibody (1:500, Invitrogen) for 1 hour at room temperature. Finally, DAPI was used to locate the nuclei. Each experiment was conducted for three times.

### 2.9 Transfection

Cells were transfected with siRNA‐PTN or scrambled siRNA using Lipofectamine 2000 (Invitrogen). The siRNA sequences were listed as follows: siRNA‐PTEN (sense:5′‐GGCGUAUACAGGAACAAUATT‐3′, antisence:5′‐UAUUGUUCCUGUAUACGCCTT‐3′), and scrambled siRNA (sense:5′‐UUCUCCGAACGUGUCACGUTT‐3′, antisense:5′‐ACGUGACACGUCCGUAGAATT‐3′). PTEN expression was detected by Western blotting. Akt‐1 plasmid was obtained from GeneCopoeia, Inc. These plasmids and siRNA were subsequently transfected into the cells using the Lipofectamine 2000 reagent (Invitrogen).

### 2.10 Western blotting

Proteins expression were evaluated by western blotting as our previous studies described.^23^ Briefly, the protein concentration was detected by bicinchoninic acid disodium (BCA, Beyotime) kit. After separation by 12% SDS‐PAGE, the protein was transferred to PVDF membrane (Invitrogen). At room temperature, 5% BSA was sealed for 1 hour, diluted primary antibody was added, incubated overnight at 4°, TBST was washed three times, then 1:1000 diluted secondary antibody was added, incubated for 2 hours, washed three times in the same way, ECL chemiluminescent substrate was added to the kit instructions at night, and exposed to color. The results were observed and processed.

### 2.11 In vivo animal assay

BALB/c nude mice (6‐week‐old, female) were obtained from Beijing Vital River Laboratory Animal Technology Co, Ltd. All the care and use of animals followed the applicable international guidelines and were approved by the Ethics Committee of the First Affiliated Hospital of Harbin Medical University. For antiproliferation studies: mice were injected with TFK‐1 cells (1 × 10^7^/0.2 mL PBS) subcutaneously into the right flank for xenograft model. After 2 weeks modeling, mice were randomly divided into 2 groups: celastrol (2 mg/kg) or PBS every 2 days (i.p.). Tumor size was measured every 7 days using calipers. (length × width^2^)/2 was used to analyzed the volume.

For antimetastasis studies: Mice were injected with TFK‐1 cells (5 × 10^6^/0.2 mL PBS) via trail vein. After 2 weeks modeling, mice were randomly divided into two groups: celastrol (2 mg/kg) or PBS every two days (i.p.). All the mice were sacrificed and the lung tissue was harvested after four weeks. All the lung nodules were counted before paraffin embedded.

### 2.12 Hematoxylin and eosin (H&E) staining

Tissues were fixed in 4% paraformaldehyde. Then the fixed tissues were dehydrated and embedded with paraffin for H&E staining.

### 2.13 Immunohistochemistry

Proteins in tumor tissues were also assessed by immunohistochemistry assay. Tissues were initially fixed and embedded. Antigen retrieval was completed in heated citrate buffer for 10 minutes. Ki‐67 (1:200), vimentin (1:200), cleaved caspase 3,9 (1:100), PTEN (1:100), and p‐AKT (1:50) were incubated overnight at 4°C. The corresponding secondary antibody was incubated for 1h. Finally, DAB (Invitrogen) was used for color development, and the slides were estimated by three independent pathologists individually.

### 2.14 Statistical analysis

Statistical analysis was used to elevate all the data. Student's *t* test or one‐way ANOVA were used for the two groups or more than two groups comparison, respectively. *P* < .05 was considered statistically significant, and *P* < .001 was highly considered significant.

## 3 RESULTS

### 3.1 Celastrol inhibits the proliferation of CCA cells

**Figure 1:**
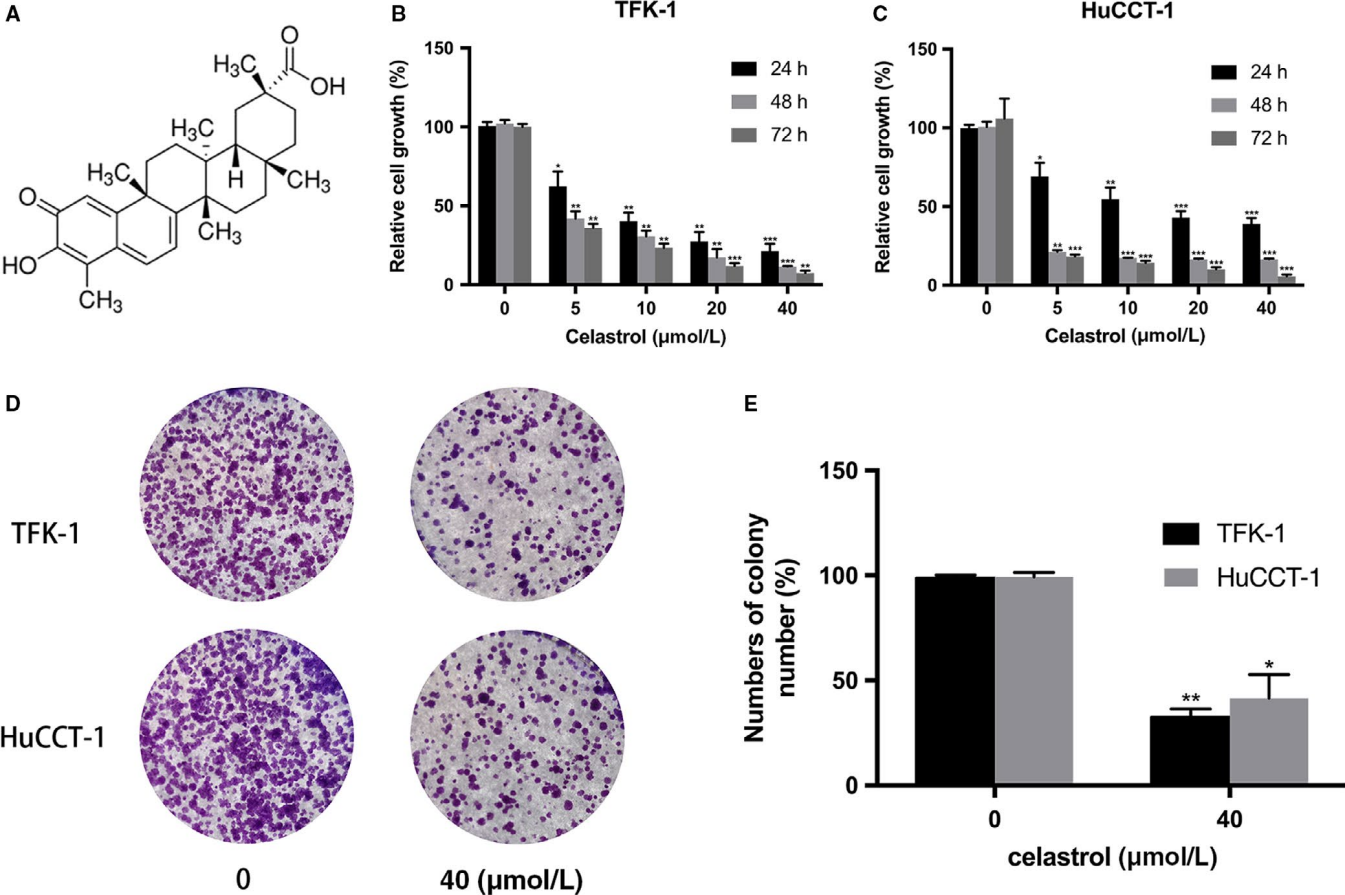
The effects of celastrol on CCA cells viability. A, The chemical structure of celastrol. B and C, TFK‐1 and HuCCT‐1 cells were treated with celastrol (0, 5, 10, 20, or 40 μmol/L) for indicated time (24, 48, or 72 h). The cell viability was analyzed using CCK‐8 assay. D and E, The numbers of colonies were counted. *P < .05, **P < .01, or ***P < .001 vs control group

We initially examined the inhibitory effects of celastrol (Figure 1A showed the chemical structure^24^ on the proliferation of TFK‐1 and HuCCT‐1 cells. Cells were incubated with celastrol for indicated time. Subsequently, cell viability was determined using CCK8 assay. We found that celastrol suppressed the viability of TFK1 and HuCCT‐1 in a dose‐ and time‐dependent manner (Figure 1B,C). To further investigate the long‐term effects of celastrol, cells were incubated with celastrol at the concentration of 40 µmol/L for 14 days, and the colony formation was performed. As Figure 1D,E showed, the number of colonies in the experimental groups were significantly lower than the control groups. These results indicated that celastrol inhibits CCA cells proliferation.

### 3.2 Celastrol triggers apoptosis in CCA cells

**Figure 2:**
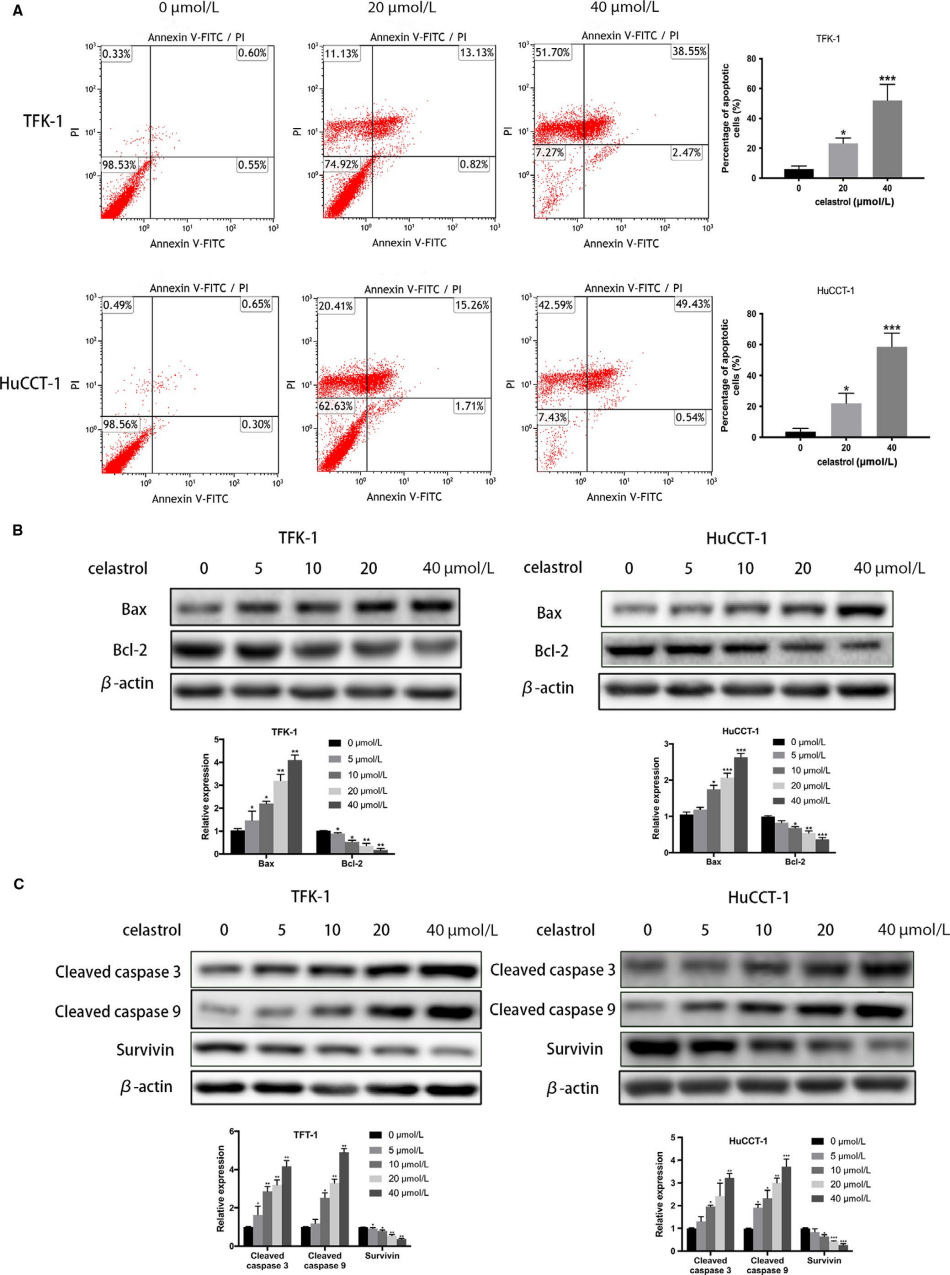
Celastrol‐induced CCA cell apoptosis. Cells were incubated with celastrol (0, 20, or 40 µmol/L) for 24 h. A, The apoptotic effect was analyzed via flow cytometry. B and C, Western blotting was performed to measure the degree of Bax, Bcl‐2, cleaved Caspase3, cleaved Caspase9, and Survivin. *P < .05, **P < .01 or ***P < .001 vs control group

To examine whether the antiproliferative effect was resulted from apoptosis induction, we performed apoptosis assay. After incubated with celastrol (0, 20 or 40 µmol/L), TFK‐1 and HuCCT‐1 cell lines were analyzed by flow cytometry (FCM) analysis using Annexin V/PI assay kit. Figure 2A showed that the apoptotic rate of TFK‐1 and HuCCT‐1 cell lines were increased in response to treatment with celastrol in a dose‐dependent manner.

Furthermore, western blotting was performed to assessed the important signaling proteins involved in celastrol‐induced cell apoptosis. As shown in Figure 2B, celastrol significantly upregulated Bcl‐2 associated X protein (Bax) expression and downregulated B cell lymphoma 2 protein (Bcl‐2) expression in a dose‐dependent manner. Thus, the Bax/Bcl‐2 ratio was increased obviously. We also evaluated the Cleaved caspase 3, 9, and Survivin protein expression. The increased Cleaved caspase 3, 9 were observed according to the data. (Figure 2C). All these data suggested that celastrol activates CCA cells apoptosis through caspase‐dependent pathway.

### 3.3 Celastrol inhibits migration and invasion of CCA cells

**Figure 3:**
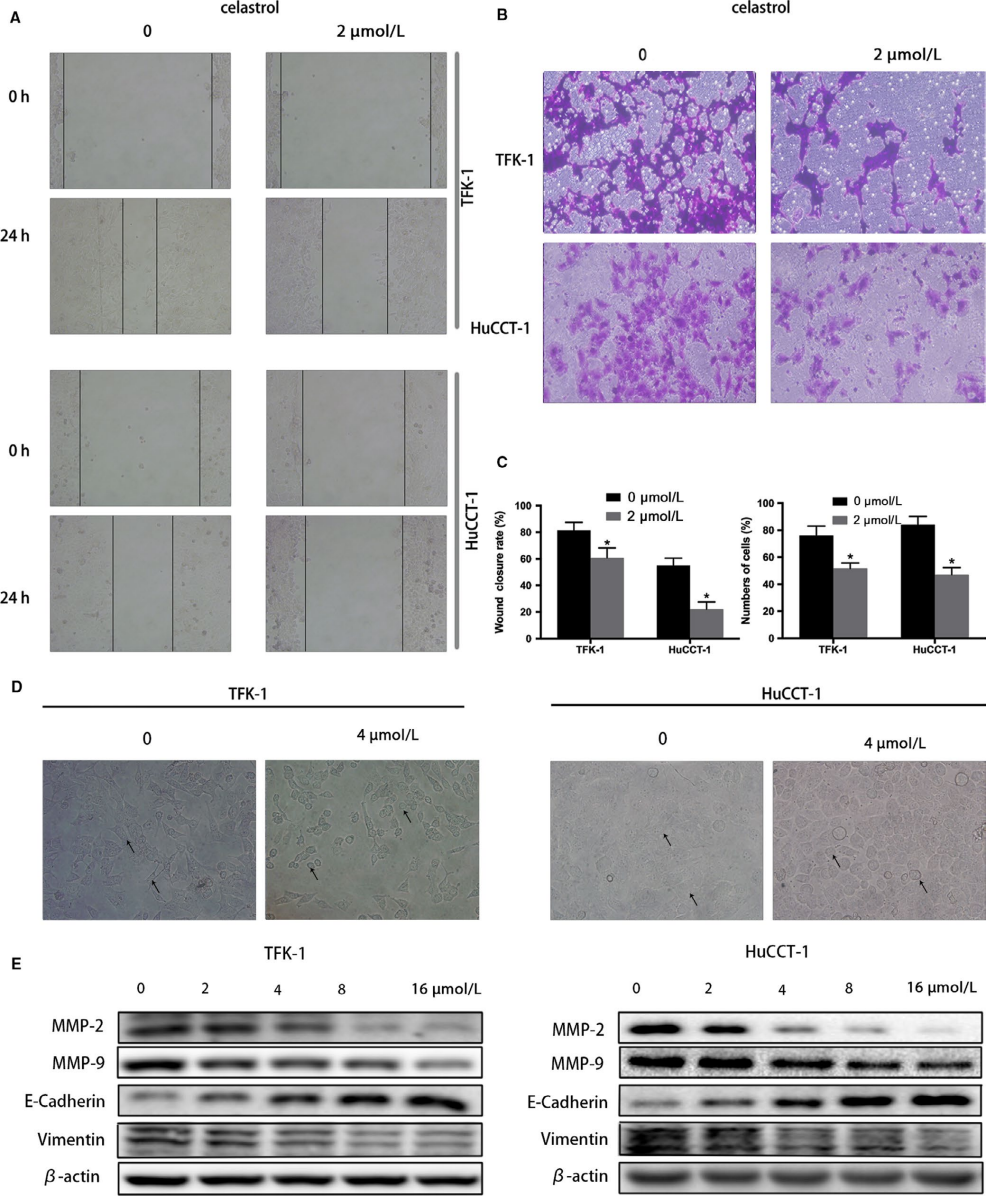
Celastrol inhibits CCA cells migration and invasion. Cells were treated with certain concentrations of celastrol. A, Wound healing assays were performed to determine the wound closure rate after cell layer was scratched. The micrographs represent cell layers before and after scratches, respectively. (200 × magnification). B, The invasive ability was examined by transwell chamber assay. The micrographs represent invasive cells on the other side of the membrane. (200 × magnification). C, Represents the statistical results of wound healing and invasion assays. D, Shows the effect of celastrol on morphological changes. E, Shows the analysis of MMP‐2, MMP‐9, E‐Cadherin, and vimentin by western blot. *P < .05 vs control group

To determine whether celastrol could inhibit migration and invasion of CCA cells, wound healing was initially performed after cultivation with celastrol for 24 hours (a low concentration that did not induce cell apoptosis). According to the data in Figure 3A, the celastrol treated cells exhibited obviously delays in wound closure. Celastrol inhibited TFK‐1 and HuCCT‐1 cells wound healing by an average of 66% and 25%, respectively, comparing to the untreated cells. Subsequently, invasion assay was performed. As data showed, the number of invaded cells was obviously decreased after celastrol incubation (Figure 3B,C).

Epithelial‐to‐mesenchymal transition (EMT) act as a primordial role in cell migration and invasion of cancer cells.^25^ As shown in Figure 3D, celastrol (0 or 4 µmol/L) changed TFK‐1 and HuCCT‐1 cells morphology to round, epithelioid phenotype from fibroblast‐like mesenchymal phenotype. We next assessed the EMT‐related proteins, including matrix metalloproteinases (MMPs), E‐cadherin, and vimentin, which promote tumor cell invasion.^26^ As expected, western blotting in Figure 3E suggested that MMP 2, 9, and vimentin proteins expression were obviously decreased, nevertheless the E‐cadherin was upregulated after celastrol incubation. All the data demonstrated that celastrol inhibits CCA cell migration and invasion through inhibiting CCA cell EMT change.

### 3.4 Celastrol inhibits PI3K/AKT signaling pathway in CCA cells

**Figure 4:**
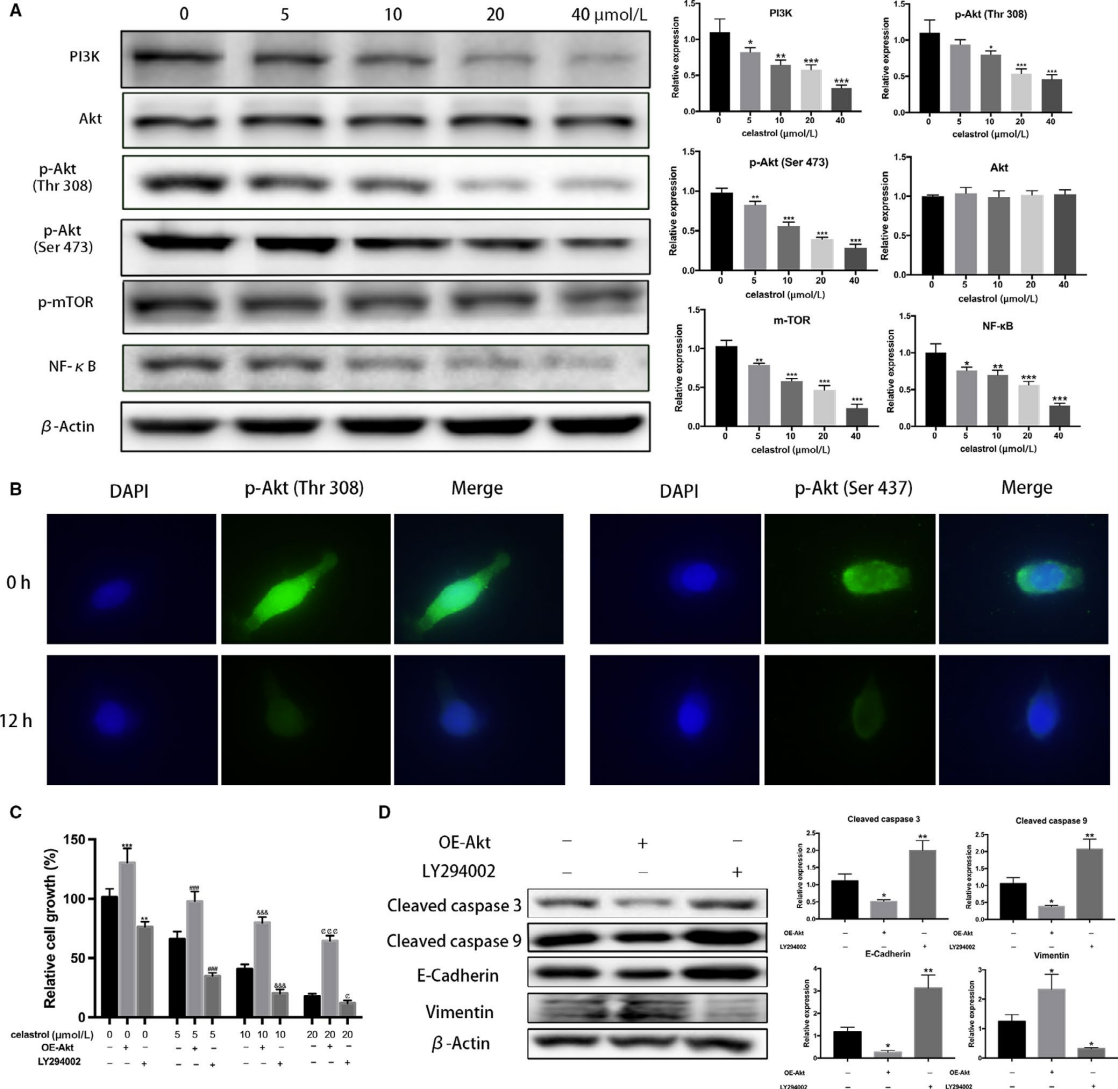
Celastrol‐induced PI3K/Akt signaling pathways blockage. A, Representative images of relative proteins expression in response to celastrol treatment in TFK‐1 cells by western blotting assay. B, p‐Akt levels were assessed using fluorescence microscopy. Original magnification: 400×. C, TFK‐1 cells were divided into Akt overexpression and low‐expression groups by cell transfection and LY294002 treatment at different celastrol concentrations. Cell viability in each group is on shown. D, Related proteins were assessed by western blotting in each group. *P < .05, **P < .01 or ***P < .001 vs control group. ###*P* < .001 vs control group. ^&&&^ *P* < .001 vs control group. ^¢^ *P* < .05 or ^¢¢¢^ *P* < .001 vs control group

Aberrant expression of PI3K/Akt signaling promotes the development of CCA oncogenesis, proliferation, and metastasis.^27^ To investigated whether the celastrol‐induced cell changes was related to the PI3K/Akt signaling, several key members were analyzed by western blotting in TFK‐1 cells. As Figure 4A,B showed, PI3K, p‐Akt, p‐mTOR, and NF‐κB expression were significantly decreased after celastrol treatment. In addition, Akt phosphorylation was observed to be inhibited at Thr 308 and Ser 473 sites by celastrol. Consistently, a significant decrease in p‐Akt was also examined by immunofluorescence assay in TFK‐1 cells (Figure 4C).

To further elucidate the role of PI3K/Akt pathway in celastrol inhibiting CCA development, we initially pretreated TFK‐1 cells with celastrol at different concentrations (0, 5, 10, and 20 µmol/L), then activated or inactivated the pathway by Akt1 transfection and LY294002, respectively. Cell viability was elevated by CCK‐8 assays. Data showed that overexpression of Akt reverse the celastrol‐induced TFK‐1 cells viability inhibition. Nevertheless, LY294002 treatment resulted in a more inhibition compared to the control groups. (Figure 4C) Additionally, related apoptosis and EMT proteins were assessed by western blotting following celastrol treatment at the concentration of 20 µmol/L. Compared with the cells only treated with celastrol, the apoptosis‐related proteins were reduced after Akt‐1 plasmid transfection, but decreased after LY294002 treatment. Meanwhile, EMT‐related proteins increased after Akt‐1 plasmid transfection, but decreased after LY294002 treatment. (Figure 4D) Our data indicated that the blockage of PI3K/Akt signaling is likely responsible for the proliferation, migration, and invasion of CCA cells.

### 3.5 Celastrol elevates PTEN expression and inhibits PI3K/AKT signaling pathway

**Figure 5:**
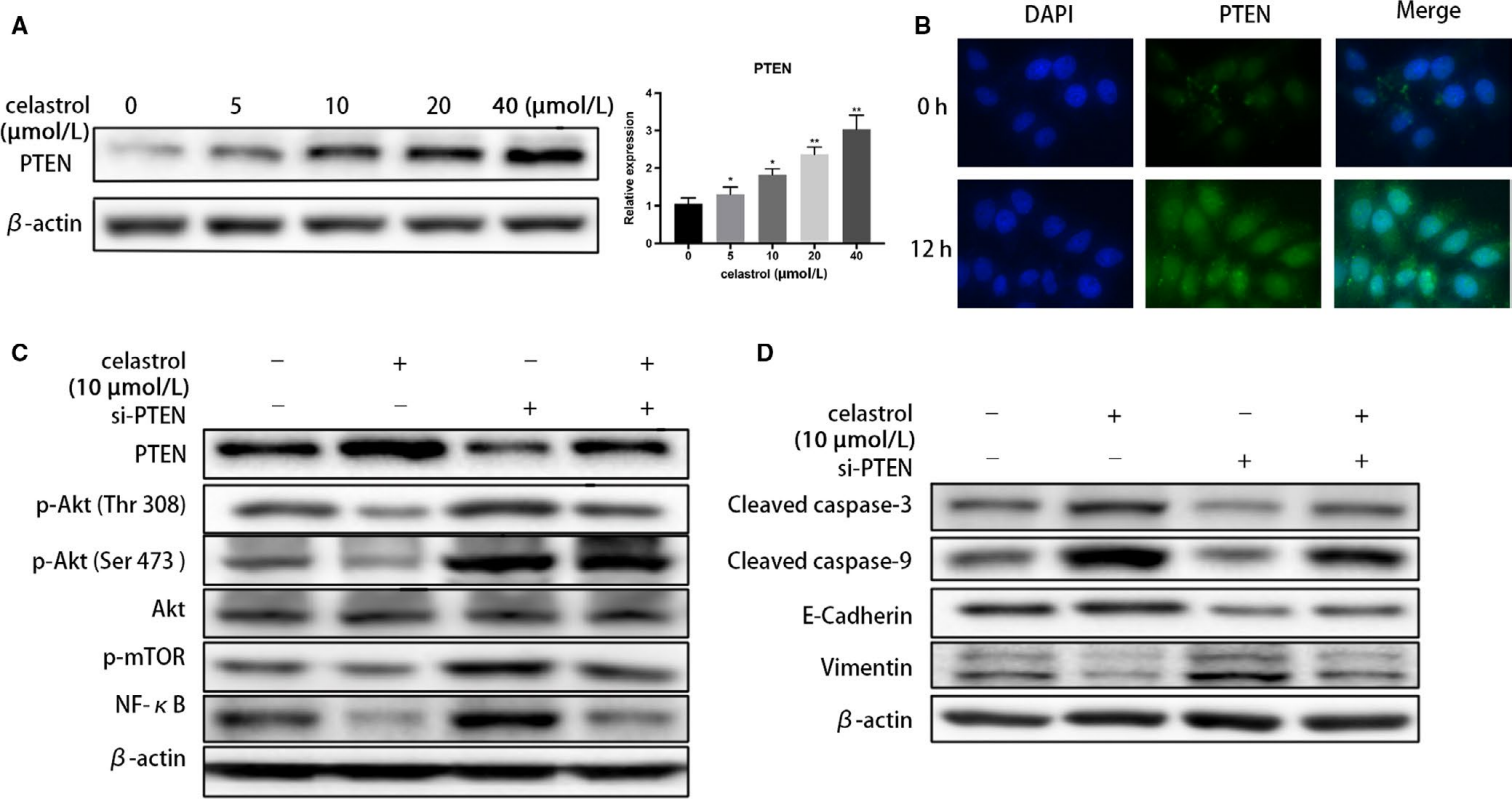
Celastrol elevates PTEN expression in CCA cells. Following indicated concentrations of celastrol treatment, A, PTEN expression was evaluated via western blotting in TFK‐1 cells. B, PTEN levels were assessed using fluorescence microscopy in TFK‐1 cells. Original magnification: 400×. C and D, After specific siRNA‐PTEN transfection in the TFK‐1 cells, related proteins were evaluated via western blotting. *P < .05 or **P < .01 vs control group

Numerous studies have shown that phosphatase and tensin homolog (PTEN) mutations occur in CCA patients, and PTEN acts as a negative regulator of PI3K/AKT signaling pathway in CCA cells. We hypothesized that celastrol elevated PTEN expression, and thus inhibited PI3K/Akt activation. As expected, the PTEN expression was observed significantly upregulated in TFK‐1 cells by western blot, following 24 hours of celastrol treatment (Figure 5A). Additionally, immunofluorescence also conformed the increased PTEN expression by celastrol (Figure 5B). To further verify the hypothesis, specific siRNA‐PTEN was transfected into TFK‐1 cells and the western blotting assays were performed. Data showed this generated knockdown of PTEN‐induced upregulation of p‐Akt (Thr 308), p‐Akt (Ser 473), p‐mTOR, and NF‐κB was reversed by celatrol treatment. (Figure 5C) Furthermore, the apoptosis and EMT‐related proteins were detected changed after PTEN knockdown by western blotting. As expected, the changes were consistent with the PI3K/Akt pathway (Figure 5D). Collectively, our data suggested that celastrol induced antitumor activity through upregulating the PTEN expression in CCA cells.

### 3.6 Celastrol inhibits CCA cells proliferation and lung metastasis in MICE

**Figure 6:**
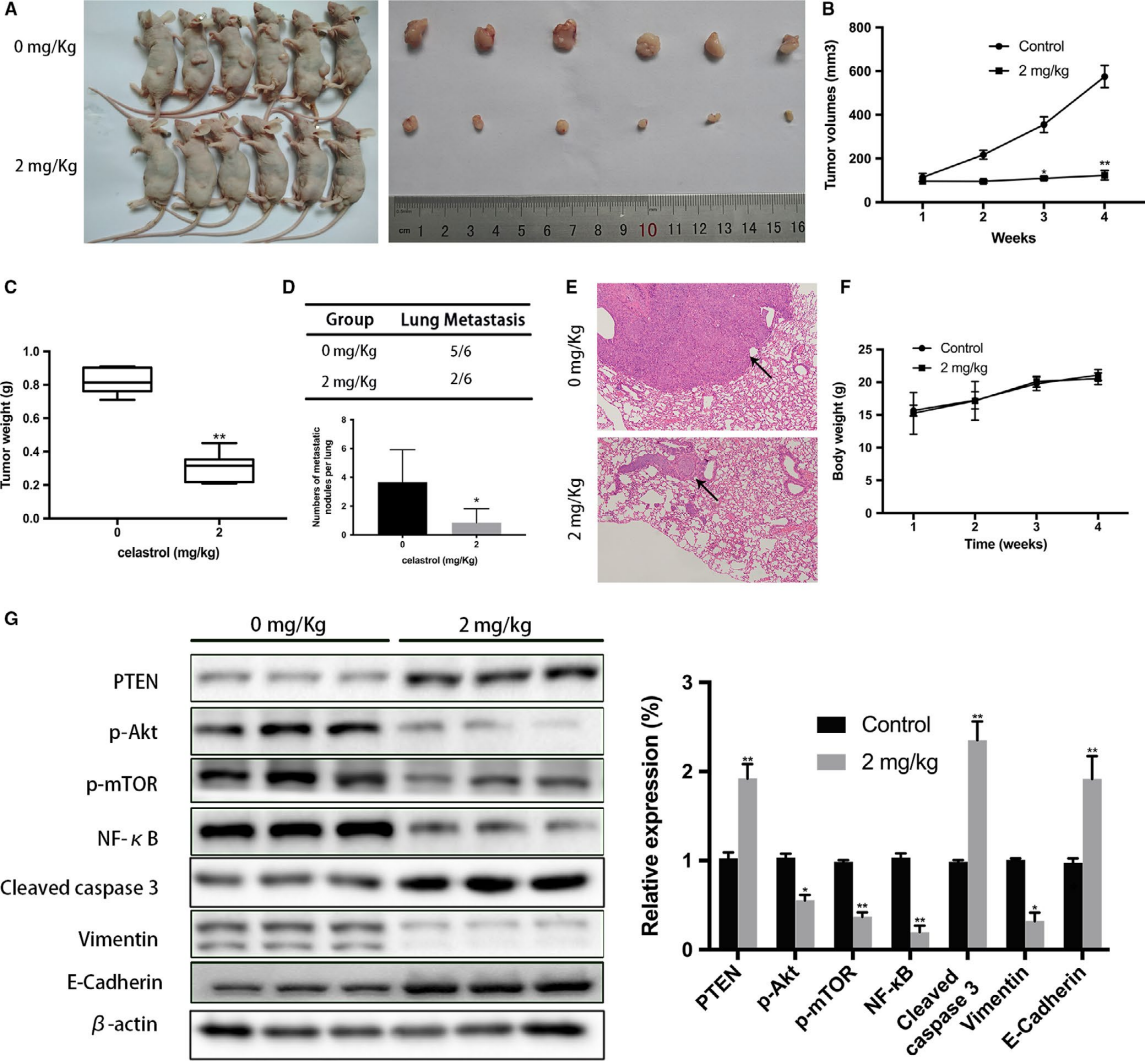
Celastrol inhibited CCA cells growth and metastasis in vivo A, Tumors were extracted from the two groups and the photos are shown. B, Represents the changes of tumor volume. C, Represents tumor weight's statistical analysis. D, Incidence of lung metastasis and numbers of metastatic nodules per lung are shown. E, Representative photos of lung metastatic nodules (100 × magnification) are shown. F, Statistical analysis of body weight. G, Related proteins expression was analyzed. *P < .05 or **P < .01 vs control group

We initially determined the antiproliferation efficiency of celastrol using a subcutaneous xenograft mice model. 12 mice were initially injected subcutaneously with TFK‐1 cells for 2 weeks, and the tumor volume had reached to a mean size of 115 mm^3^ followed by treatment of vehicle control or celastrol (2 mg/kg) every 2 days. Figure 6A presented the tumors harvested from the two groups. In addition, Figure 6B showed the tumors volume change dunning this experimental period, and data showed that tumors grew continuously in the celastrol group, whereas tumor growth was significantly suppressed in vehicle control group. Additionally, the mean weight of tumors in the celastrol group was 0.29 g, whereas that of tumors in the control animals was 0.81 g. (Figure 6C).

We subsequently determined the antimetastasis efficiency of celastrol using a lung metastatic mice model. 12 mice were injected with TFK‐1 cells via the trail vein for 2 weeks followed by celastrol treatment. After 4 weeks, the number of nodules and incident rate were drastically decreased with celastrol treatment (Figure 6D) compared to the control treatment. Furthermore, HE staining results also showed that the lung metastasis nodules in mice treated with celastrol were significantly smaller than those of mice in the control group. (Figure 6E) Importantly, to evaluate potential adverse effect of celastrol, Mice (n = 10) were treated with PBS or celastrol (2 mg/kg) every 2 days for 4 weeks. Data showed no apparent body weight changes or toxicity‐related events were observed in the celastrol group (Figure 6F).

**Figure 7:**
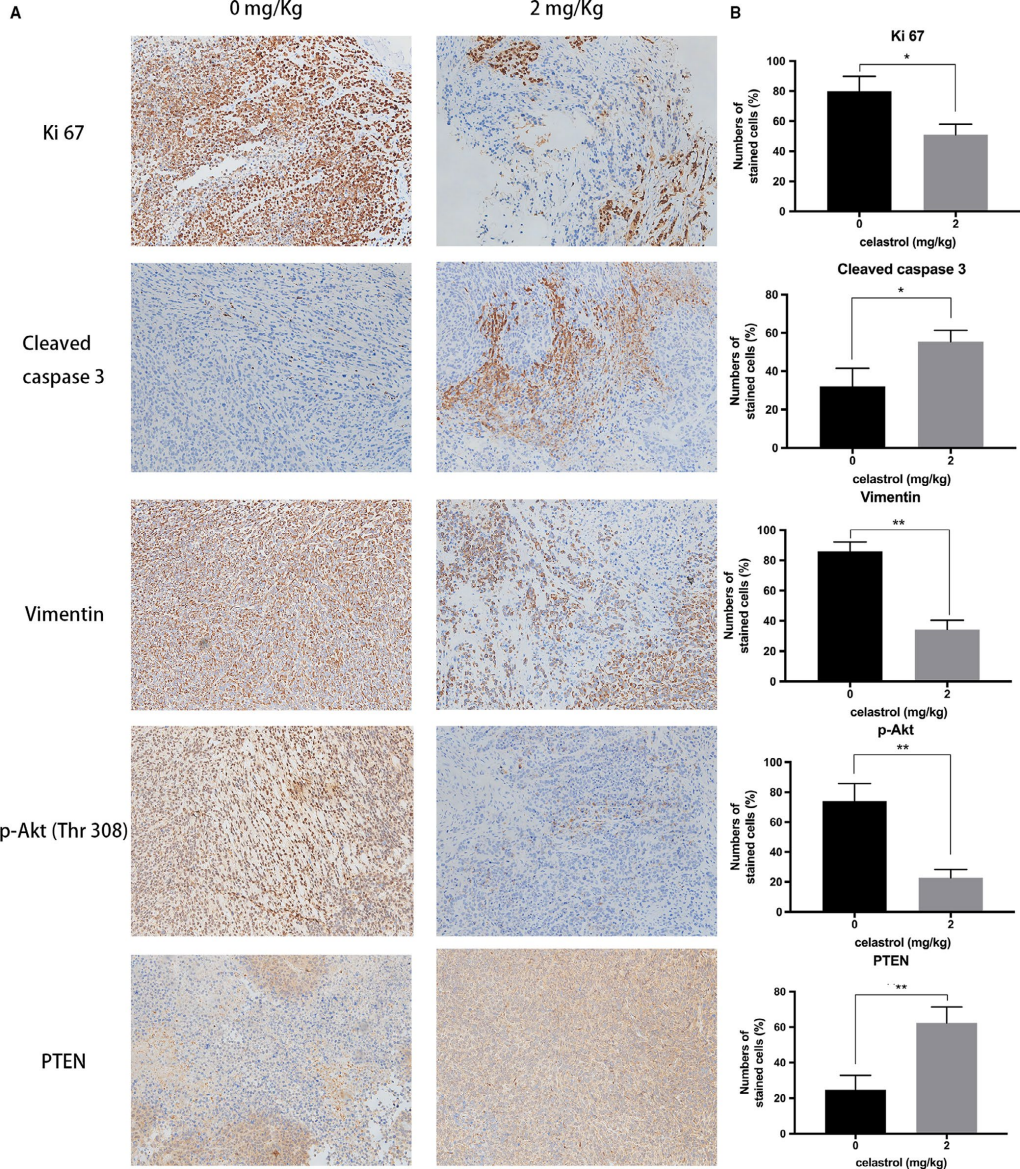
Immunohistochemical characterization of tumor tissues. A, Immunohistochemical analyses of Ki 67, cleaved caspase 3, vimentin, p‐AKT, and PTEN. Note the proliferation activity in tissue by Ki67‐positive tumor cells, apoptosis by cleaved caspase‐3, and EMT by vimentin (200 × magnification). B, The levels of relative proteins were analyzed. *P < .05 or **P < .01 vs control group

Furthermore, PI3K/PTEN/Akt signaling‐related proteins expression was analyzed in the excised tumor tissues using Western Blot. As expected, the p‐AKT and vimentin expression were decreased, whereas the cleaved caspase‐9 and PTEN expression were increased according to the data. (Figure 6G) A immunohistochemistry assay further conformed the increased expression of PTEN and cleaved caspase‐3, and an apparent decreased expression of Ki 67, p‐AKT, and vimentin (Figure 7). Collectively, in vivo experiments conformed celastrol inhibited the tumor growth and lung metastasis with no serious side effects.

## 4 DISCUSSION

Limited clinical option for treatment remains an aggressive challenge for the patients with CCA, especially in advanced stage. Increasing studies suggest that natural compounds may be a new option for the development of potent cancer treatments.^28^, ^29^, ^30^ Celastrol, a component of traditional Chinese medicine, has been found widespread application, and exhibits many biological activities. However, whether celastrol could suppress CCA development remain unknown. In this study, our results demonstrated celastrol exhibited effects on CCA cells growth, migration, and invasion. The proapoptosis effect was also observed in the CCA cells by celastrol. Additionally, celastrol inhibited CCA cell proliferation and metastasis without apparent potential adverse effects in vivo. Further study revealed mechanism that celastrol induced the inhibitory effects through PTEN/PI3K/Akt signaling axis in vitro and in vivo.

Proapoptosis activity acts as one of the most common antitumor mechanisms to control tumor proliferation, and numerous studies proved that mitochondrial apoptosis plays a vital role in human cholangiocarcinoma.^31^ The mitochondrial apoptosis process can be described briefly as follows: Under the action of various apoptotic signals, mitochondrial permeability transition pore (MPTP) irreversibly opens excessively, mitochondrial transmembrane potential disintegrates, respiratory chain uncoupling, mitochondrial matrix osmotic pressure increases, intima swelling, cytochrome C (Cytc), and other apoptotic active proteins located in the mitochondrial membrane gap are released into the cytoplasm. In the presence of ATP/dATP, Cytc, and apoptotic protease activator‐1 (Apaf‐1) form a polymer complex, which activates the precursor of Caspase‐9, leading to the activation of downstream effector Caspase‐3, and cutting the substrate to induce apoptosis.^32^ Numerous of evidence demonstrated that celastrol inhibit cancer cells proliferation through proapoptosis mechanism, including oral cancer, lung cancer, breast cancer, and colon cancer.^33^, ^34^, ^35^, ^36^ In our study, western blotting and IF assays demonstrated that cleaved caspase‐3,9 were significantly increased when compared with the control groups, supporting that proapoptosis activity was induced after the celastrol treatment in CCA cells. Furthermore, Bax/Bcl‐2 is associated with cell apoptosis according to numerous studies.^37^ As our study showed, celastrol was indeed able to promote apoptosis by regulating the Bax/Bcl‐2 ratio, indicating that celastrol‐induced cell death by the mitochondria‐mediated intrinsic apoptosis pathway.

EMT is an important molecular mechanism of invasion and metastasis of malignant tumors.^34^ A large amount of evidence from experimental and clinical researches have highlight the important role of EMT in CCA metastasis.^38^ Celastrol has been reported to exhibit inhibitory effects on the progression of EMT in various of cancers.^39^, ^40^, ^41^ Our present studies proved that celastrol substantially inhibited the levels of epithelial marker E‐cadherin and decreased the levels of mesenchymal markers N‐cadherin and vimentin. All the results supported that celastrol could inhibit the CCA cell EMT progression, thus suppressing CCA growth and metastasis.

The PI3K/Akt pathway acts as a vital role in tumor oncogenesis and development, including cell viability, autophagy, migration, metastasis, angiogenesis, and drug resistance.^42^ Recent studies discovered the frequent aberrations of PI3K/Akt signaling in CCA development.^43^ Schuurbiers OC et al reported that p‐Akt is associated with antiapoptosis through regulating the proapoptotic protein expression.^44^ Importantly, mTOR and NF‐κB are key downstream effectors of the PI3K/Akt pathway.^45^ The activated mTOR is associated with cell viability and apoptosis, whereas the NF‐κB is with cell behavior.^46^, ^47^ To gain insight into the underlying mechanisms by celastrol, we evaluated the PI3K/Akt/mTOR and NF‐κB signaling‐related molecule proteins expression. As respected, the decreased PI3K expression was observed significantly by western blotting. Although the total levels of Akt was not altered, the NF‐κB, p‐Akt, and p‐mTOR expression was inhibited by celstral treatment in TFK‐1 cells. In our further experiments, upregulated and downregulated PI3K/Akt pathway reversed or accelerated the celastrol‐induced CCA cells proliferation and metastasis, respectively. All the data supported that celastrol induced CCA inhibition by the mechanism of PI3K/Akt/mTOR and NF‐κB signaling axis.

PTEN (phosphatase and tensin homologue) is known as a tumor suppressor, which was frequently lost or mutated in various human malignances.^48^, ^49^ Clinicopathologic data demonstrated that downregulated levels of PTEN were found in patients with T classification and stage grouping. Additionally, the increased levels of PTEN correlates with longer survival.^50^ Mechanically, PTEN activates PIP3 to PIP2, and then inhibits PI3K/AKT signaling pathway which meditates embryonic development, cell viability, apoptosis, differentiation, migration, and metastasis.^51^, ^52^, ^53^ The present data indeed proved that celastrol induced PTEN upregulation and significantly inhibited PI3K/AKT signaling pathway in CCA cells. In the additionally loss‐of‐function studies, we downregulated the expression of PTEN in by transfection with siRNA against PTEN, and found that blockage of PTEN‐induced significant upregulated PI3K/AKT signaling pathway compared to the control group. These results suggest that the celastrol‐induced blockage of the PI3K/AKT pathway is due to the upregulation of PTEN. However, PTEN knockout mice might a better option for conforming the results, and it would be used in our further studies.

## 5 CONCLUSION

**Figure 8:**
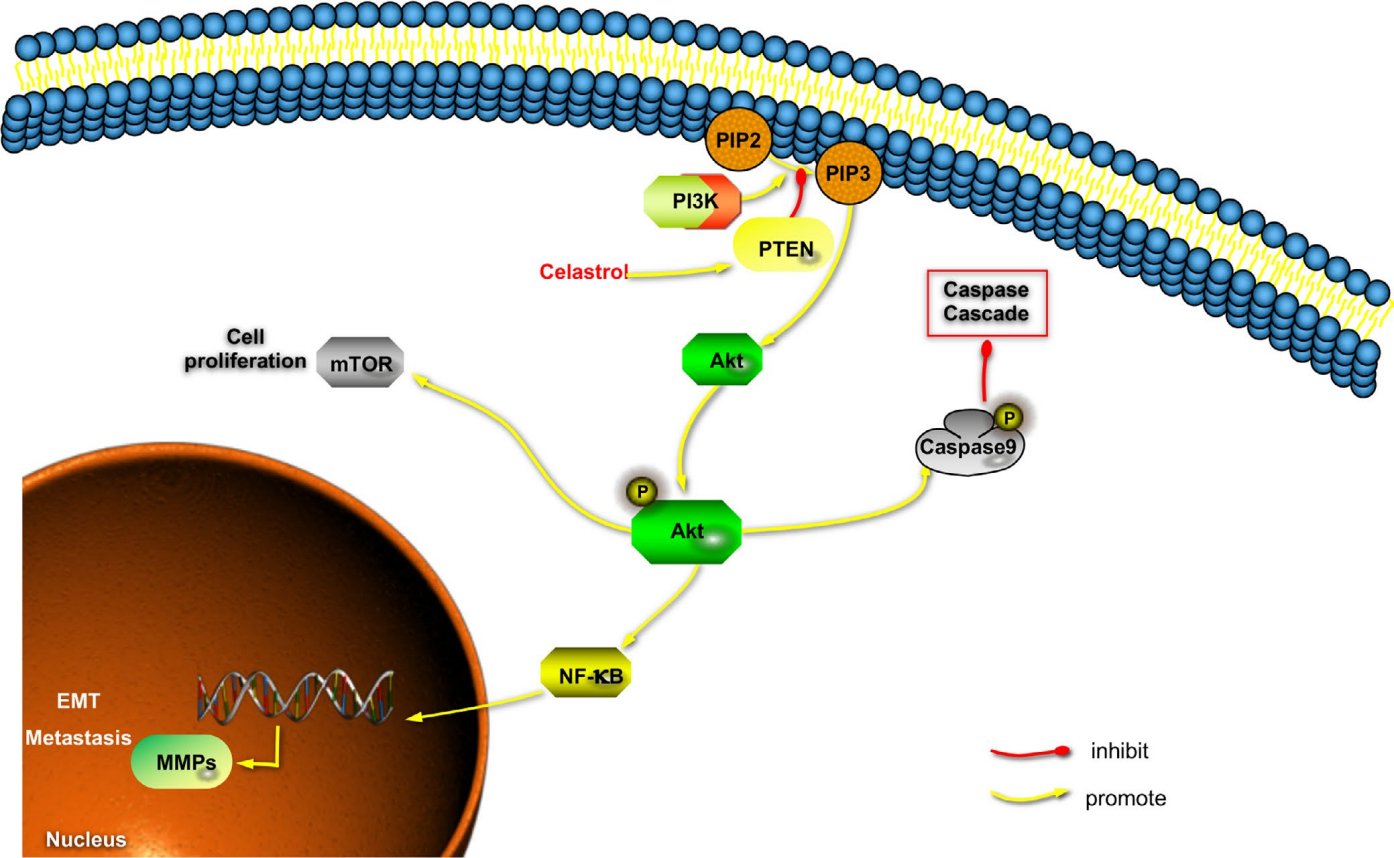
Diagram illustrating the proposed effects of celastrol on PI3K/Akt/mTOR and NF‐κB signaling pathways

In summary, we report a novel component that exhibits a significant effect on CCA cells growth and metastasis in vivo and in vitro. Further mechanism studies demonstrated that the inhibitory effects are attributed to the induction of apoptosis, and the blockade of migration and invasion might be regulated by perturbations in the PI3K/Akt signaling. Furthermore, and these perturbations were regulated by the drug induce‐PTEN upregulation (Figure 8). Therefore, our study provides preclinical evidence for the CCA treatment with celastrol as a new strategy.

## CONFLICTS OF INTERESTS

The authors declare no conflict of interest.

## AUTHOR CONTRIBUTIONS

Biqiang Zhu designed and performed the experiments. Yunwei Wei analyzed the data. Biqiang Zhu wrote the manuscript.
